# Risk Factors and Spatio-Temporal Patterns of Human Rabies Exposure in Northwestern Tigray, Ethiopia

**DOI:** 10.5334/aogh.2518

**Published:** 2019-09-09

**Authors:** Gebreyohans Gebru, Gebremedhin Romha, Abrha Asefa, Haftom Hadush, Muluberhan Biedemariam

**Affiliations:** 1 Department of Animal Sciences, College of Agriculture, Aksum University, Shire Campus, Shire, Tigray, ET; 2 Department of Animal Production and Technology, College of Agriculture and Environmental Science, Adigrat University, Adigrat, Tigray, ET; 3 Department of Geography and Environmental Studies, Adigrat University, Adigrat, ET; 4 Department of Biomedical Science, College of Health Science, Aksum University, Aksum, Tigray, ET; 5 Department of Soil Resource and Watershed management, College of Agriculture, Aksum University, Shire Campus, Shire, Tigray, ET

## Abstract

**Background:**

Rabies is a neglected tropical disease, which is economically important with great public health concerns in developing countries including Ethiopia. Epidemiological information can play an important role in the control and prevention of rabies, though little is known about the status of the disease in many settings of Ethiopia. The present study aimed to investigate the risk factors and spatio-temporal patterns of human rabies exposure in Northwestern Tigray, Ethiopia.

**Methods:**

A prospective study was conducted from 01 January 2016 to 31 December 2016 (lapsed for one year) at Suhul general hospital, Northern Ethiopia. Data of human rabies exposure cases were collected using a pretested questionnaire that was prepared for individuals dog bite victims. Moreover, GPS coordinate of each exposure site was collected for spatio-temporal analysis using hand-held Garmin 64 GPS apparatus. Later, cluster of human rabies exposures were identified using Getis-Ord G_i_* statistics.

**Results:**

In total, 368 human rabies exposure cases were collected during the study year. Age group of 5 to 14 years old were highly exposed (43.2%; 95% CI, 38.2–48.3). Greater number of human rabies exposures was registered in males (63%; 95% CI, 58.0–67.8) than females (37%; 95% CI, 32.1–42.0). Residents of rural (85.6%; 95% CI, 81.6–88.8) areas were at greater risk to rabies than urban residents (14.4%; 95% CI, 11.2–18.4). Higher proportion of human rabies exposures were caused by unprovoked (96.5%; 95% CI, 94.0–98.0) and unvaccinated (85.9%; 95% CI, 81.9–89.1) dogs. All rabies exposures were exclusively caused by dog bites and the majority of them (80.4%; 95% CI, 76.0–84.2) were caused by stray dogs. Results of spatio-temporal analysis showed that Asgede Tsimbla, Endaselassie and Laelay Adiyabo districts experienced the highest burden of rabies exposure; identified as hot spots. Strong peaks of human rabies exposure occurred between March and July months.

**Conclusion:**

The present study provided basic epidemiological information on the potential risk factors associated with human rabies exposure. Moreover, our findings provided basis for understanding the spatio-temporal patterns of human rabies in Northwestern Tigray districts for the first time.

## Background

Rabies is a fatal viral disease that affects the central nervous system of all warm-blooded animals, including humans [1,2,3]. Domestic dogs have remained as the most important vectors worldwide, causing greater than 95% of all human rabies cases [4,5,6,7,8]. Rabies is almost 100% fatal once the symptoms of the disease develop [2,9,10]. However, effective vaccines to prevent rabies are widely available for humans and dogs [10,11]. Although most developed countries have eliminated the disease, rabies is yet the most important deadly disease in developing countries with low public health and veterinary service settings [12].

Rabies is estimated to cause 59,000 human deaths per year globally. It also causes about 3.7 million disability-adjusted life years (DALYs) burden and 8.6 billion USD economic losses per year in the world [12]. Elimination of dog-transmitted rabies as a public health problem has been documented to be feasible by vaccinating dogs and providing post-exposure prophylaxis (PEP) to humans until dog rabies is eliminated [3,13,14]. However, this should be complemented with mandatory legislations for registration, certification and regular vaccination of owned dogs; depopulation of stray dogs; creating public awareness as well as provision of easily accessible and affordable post exposure vaccines for humans [3].

Most human deaths due to rabies occur in developing countries of Asia and Africa [5]. Of these, Ethiopia is one of the worst affected [12]. Domestic dogs are the principal reservoirs of rabies in Ethiopia [8,15]. Data on dog demography is not available and policies for controlling dog breeding and population are also lacking in the country [9]. Besides, dog management is poor and anti-rabies dog vaccination is often available to owned dogs found in urban settings [16]. Large population of dogs combined with poor dog management contributes to the high endemicity of canine rabies in the country [15]. Severe under-reporting of human rabies cases and lack of record keeping have also been reported in Ethiopia [17]. This has resulted in underestimating the actual burden of the disease and made it less prioritized for control and prevention. Besides, a substantial number of exposed to rabies prefer to seek medical care from traditional healers rather than visiting health care centers [18,19]. Efforts aiming at rabies control are fragmented due to weak coordination between the veterinary and public health sectors. The supply of anti-rabies dog vaccines is also limited and irregular [8]. Even in situations when anti-rabies vaccines are available, owners are not willing to vaccinate their dogs due to lack of awareness [15].

Rabies is an endemic and economically important disease with great public health concern in Northwestern Tigray. A retrospective study conducted at Suhul hospital, located in Northwestern Tigray, reported that the annual incidence of human rabies was much higher than previously reported in other studies conducted at different settings of Ethiopia [8]. Epidemiological information such as analysis of risk factors and integrating spatio-temporal data can play an important role in the prevention and control of rabies. Spatio-temporal human rabies distribution has become easier with the development and improvement of Geographic Information Systems (GIS), software systems that visually represent information spatially and non-spatially on maps. However, little is known about the spatio-temporal distribution of disease in Northwestern Tigray, which hinders the implementation of cost-effective disease prevention and control measures. The objectives of this research were, therefore, to give insights into the risk factors and spatio-temporal patterns of human rabies exposure in Northwestern Tigray, Ethiopia.

## Methods

### Description of the study area

**Figure 1:**
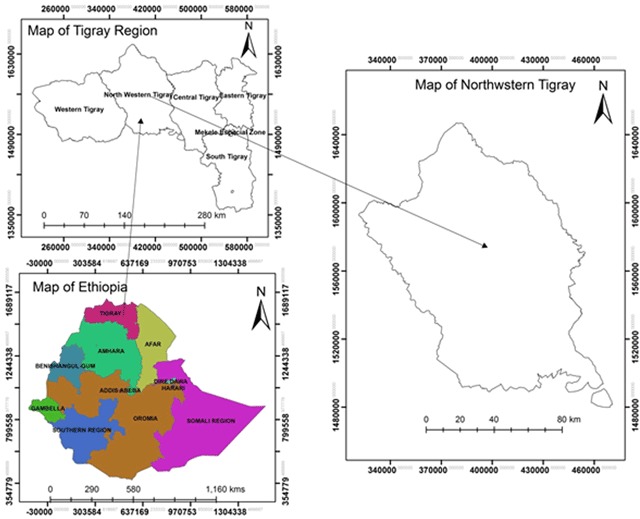
Location map of the study area.

Health facility study was conducted at Suhul general hospital based on human PEP data. Dog bite victims admitted in the hospital were from eight districts of Northwestern Tigray namely: Shire Endaselassie, Laelay Adiabo, Tselemti, Tahtay Adiabo, Asgede Tsimbla, Sheraro, Medebay Zana and Tahtay Koraro. The hospital is located at Shire Endaselassie town in Northwestern Tigray (Figure 1). Northwestern Tigray is a zonal administration with an altitude ranges from 645 to 2852 meters above sea level. The total area of the zone (in Ethiopia, zone is a large political administrative unit next to regional state) is estimated to be 18,325 km^2^ with an average annual rainfall of 878 mm, which mainly occurs between June and September. Average annual temperature of the area varies from 18 to 34.6°C. More than 40% of Northwestern Tigray is estimated to be covered with forests. It is also one of the areas of the Tigray region, where high population of livestock are found. Kafta Sheraro national park, which is the home for 95 avian and 42 mammalian wildlife species, are also found in this zone.

### Identification of human rabies exposure

A prospective study was conducted from 01 January 2016 to 31 December 2016 (lapsed for one year) at Suhul general hospital. Dog bite victims admitted to the hospital were purposively selected for the study. As practice, if a bite victim is admitted to the hospital, his/her wound would be treated, and allowed to take the Tetanus antitoxin (TAT) based on the degree of bite/puncture. Meanwhile, the dog inflicting the bite is followed up and observed for 10 days. Bite victims contacted with rabies suspected dogs were diagnosed according to the guideline described elsewhere and as elaborated in Teklu et al. [8,20]. The behavioral manifestation and clinical signs of the biting dogs to categorize as rabid or non-rabid was managed based on the six criteria of rabies diagnosis in living dogs as described in Tepsumethanon et al. [21]. Some dogs also die within the 10 days observation period. Recommendations for PEP were made based on careful analysis from the history of exposure of biting dog as well as observation of classical clinical signs of the disease in the dog and/or its death. If the biting dog is categorized as rabid or had died during the observation period, the victim is assumed to be exposed to rabies virus and is allowed to receive complete dose of PEP. On the other hand, if the dog remained healthy during the observation period, the victim was not recommended to take PEP. In case of victims bitten by stray/or free roaming dogs, it was not possible to follow them. Therefore, the victims were recommended to start PEP immediately, depending on the type of exposure.

To classify human rabies exposure types (as type I, II or III), Suhul general hospital used similar protocol that was consistent with the European Centre for Disease Prevention and Control (ECDC) definition [22]. Dog bite victims, possibly exposed to rabies, included in this research were only category II (minor exposure) and category III (severe forms) exposures, recommended to receive PEP at Suhul hospital.

### Operational definitions

The following human rabies exposure operational terms were used at Suhul general hospital, as adopted from ECDC [22]:

- Category of exposure type I – Contact of intact skin with secretions or excretions of a rabid animal or human case.
- Category of exposure type II – Nibbling of uncovered skin; and minor scratches or abrasions without bleeding.
- Category of exposure type III – Single or multiple transdermal bites or scratches, licks on broken skin; Contamination of mucous membrane with saliva (i.e. licks); and Exposure to bats.
- Rabies post exposure prophylaxis (PEP) – rabies PEP was administered for category II and III exposures and 17 total doses were given through subcutaneous route around the umbilicus (14 doses for the first 14 consecutive days and three booster doses on the 10^th^, 20^th^ and 30^th^ days following the last injection). Five percent of aqueous suspension of brain tissue of sheep inoculated with fixed rabies virus, inactivated with Phenol (Fermi-Type) was used as PEP. The vaccine is produced by the Ethiopian Health and Nutrition Research Institute (EHNRI). The recommended doses were different for different age groups. Administration of rabies immune globulin (RIG) was not practiced in the hospital and there was no access for pre-exposure immunization in the study area.
- Provoked Attack – Entering an unfamiliar compound with a guard dog; stepping on or bumping into a dog; interfering in a dog fight; taking puppies from their mother; taking food from a dog or beating a dog.
- Unprovoked Attack – Being bitten by the victim’s own dog that has no prior history of dominance aggression.

### Inclusion and exclusion criteria

Dog bite victims were being judged to be exposed to rabies virus if they satisfy exposure types II and III as well as classic behavioral manifestations and clinical signs of the disease were observed in the biting dogs and/or ended fatally. However, when the victims were bitten by stray dogs, quarantine of dogs for observation of clinical signs was not manageable the victims were assumed as exposed. Individuals who were inflicted by owned or stray dogs, and categorized under exposure type I were considered as non-exposed and excluded from the research.

### Data Collection

To collect relevant information about human rabies exposure, we prepared a pretested questionnaire which was similar to that of WHO [3]. Victims suggested to be exposed to the rabies virus and included in this study were anonymized for the sake of protection of their medical confidentiality rights. Potential risk factors such as age, sex, residence, site of bite, and previous history of exposure and anti-rabies vaccination of exposed individuals were included in the data collection tool. Likewise, factors such as ownership, vaccination history and provocation of biting dogs were also incorporated. GPS coordinates of each case site were also collected for spatio-temporal analysis. GPS co-ordinates were collected with the help of Garmin 64 hand-held GPS apparatus for each rabies exposure. Data of human rabies exposures were collected and prepared in table format.

We projected the results of the population census of Northwestern Tigray in order to estimate the human population at risk of developing rabies [23]. The growth rate of the population in the study area was considered 2.5%, which is equivalent to the Tigray regional state population growth rate. During the study year, 368 human rabies exposure cases were registered and received complete doses of PEP.

### Statement of ethical clearance and informed consent

The study protocol was reviewed and approved by the scientific and ethical review committee of Aksum University, College of Health Sciences. Permission was sought from the hospital administration before data collection. Moreover, an official letter was submitted to Suhul hospital stating that the findings would be used for scientific purposes. It was also explained to all patients that the data of individuals would be anonymized and kept confidential.

### Statistical analysis

The collected data were analyzed using STATA statistical software (version 14.0, Stata Corp, college station, Texas 77845 USA). The suspected risk factors (age, sex, residence, site of bite, provocation, and vaccination status, ownership of biting dog and category of exposures) for the occurrence of human rabies exposures such as were analyzed using descriptive statistics.

### Hot spot and cold spot analysis

The table containing column of rabies exposure and co-ordinate was imported to ArcGIS (version 10.5) software and it was added to the shape file of the study area. The shape file of the study area and the points of rabies exposure were Geo-referenced in WGS_1984_UTM system along with the respective projection system so that the rabies exposure points were overlaid on the shape file of the study area for further spatial analysis. After plotting the coordinates into the map, hotspot analysis was done using a model builder tool in GIS environment.

Afterwards the Inverse ArcGIS Spatial Analyst extension was used which provides a rich set of spatial analysis and modeling tools for both raster (cell-based) and feature (vector) data. With the help of this technique, the spatial distribution human rabies exposures in raster format were produced to get every point in the study area represented. The method used in this analysis involves the Distance Weighted (IDW) Interpolation technique to predict the value of unknown points from the known points.

Later the Hot-spot analysis was carried out in order to identify the places of more and less occurrence of rabies. Hotspot analysis is done in a condition indicating some form of clustering in a spatial distribution. This analysis uses the Getis-OrdGi which can separate clusters of high values from cluster of low values. Cluster of human rabies exposures were identified using Getis-Ord G_i_* statistic. The G_i_* statistic measures the degree of spatial clustering of a local sample and how different it is from the expected value (Equation 1). It is calculated as the sum of the differences between values in the local sample and the mean, and is standardized as a z-score with a mean of zero and a standard deviation of 1:

1 {G_i^*}{\rm{ = }}\frac{{\sum\nolimits_{j = 1}^n {{w_{i,j}}{x_j} - \bar X\sum\nolimits_{j = 1}^n {w_{i,j}}}}}{{S\sqrt {\frac{{\left[ {n\sum\nolimits_{j = 1}^n {w_{i,j}^2 -} \;(\sum\nolimits_{j = 1}^n {{w_{i,j}}){^2}}} \right]}}{{n - 1}}}}}

Where *x_j_* is the attribute value for feature *j, w_ij_* is the spatial weight between feature *i* and *j, n* is equal to the total number of features equation 2 and 3.

2 \bar X = \frac{{\sum\nolimits_{j = 1}^n {{w_j}} }}{n}

3 S = \sqrt {\frac{{\sum\nolimits_{j = 1}^n {x_j^2} }}{n}} - {(\bar X)^2}

Based on this formula, statistically significant spatial clusters of high values (hot spots) and low values (cold spots) were identified. For statistically significant positive z-scores, the larger the z-score is, the more intense the clustering of high values (hot spot). For statistically significant negative z-scores, the smaller the z-score is, the more intense the clustering of low values (cold spot).

## Results

### Demographic and epidemiological risk factors

In total, 368 human rabies exposures were officially registered and followed up their PEP at Suhul hospital in 2016. All the exposures registered at Suhul hospital throughout the study year were caused by dog bites. The age group of 5 to 14 years was the most exposed (43.2%; 95% CI, 38.2–48.3). A greater number of human rabies exposures was registered in males (63%; 95% CI, 58.0–67.8) than females (37%; 95% CI, 32.1–42.0). The proportion of human rabies exposures was greater in rural (85.6%; 95% CI, 81.6–88.8) than urban residents (14.4%; 95% CI, 11.2–18.4). Inflicted individuals included in this research were only type II and III exposures. A higher proportion of human rabies exposures was caused by unprovoked dogs (96.5%; 95% CI, 94.0–98.0), and of these, the majority were unvaccinated (85.9%; 95% CI, 81.9–89.1) (Table 1). In this study, incidence of human rabies exposure was calculated to be 40 per 100,000 populations (taking 920,169 as population at risk).

**Table 1:** Characteristics of human rabies exposure cases in Northwestern Tigray, Ethiopia, 2016 (N = 368).

Risk factors		No. of human rabies exposures	%	Standard error	95% CI

Age	0–4	45	12.2	1.7	9.2–16.0
	5–14	159	43.2	2.6	38.2–48.3
	15–24	48	13.0	1.7	9.9–16.9
	25–34	42	11.4	1.6	8.5–15.1
	35–44	30	8.1	1.4	5.7–11.4
	45–54	29	7.8	1.4	5.5–11.1
	55–64	9	2.4	0.8	1.3–4.6
	65^+^	6	1.6	0.7	0.7–3.5
Sex	Male	232	63	2.5	58.0–67.8
	Female	136	37	2.5	32.1–42.0
Residence	Urban	53	14.4	1.8	11.2–18.4
	Rural	315	85.6	1.8	81.6–88.8
Site of bite	Heads	3	0.8	0.5	0.2–2.5
	Limbs	361	98.1	0.7	96.1–99.1
	Torso	4	1.1	0.5	0.4–2.9
Category of exposure	II	209	56.8	2.5	51.7–61.8
	III	159	43.2	2.5	38.2–48.3
Ownership of biting dog	Owned	72	19.6	2.1	15.8–24.0
	Stray	296	80.4	2.1	76.0–84.2
Provocation of dogs	Provoked	13	3.5	0.9	2.1–6.0
	unprovoked	355	96.5	0.9	94.0–98.0
Vaccination of dogs	Vaccinated	52	14.1	1.8	10.9–18.1
	Unvaccinated	316	85.9	1.8	81.9–89.1

CI: Confidence Interval.

### Spatial and temporal distribution of human rabies exposure in Nowrthwestern Tigray

**Figure 2:**
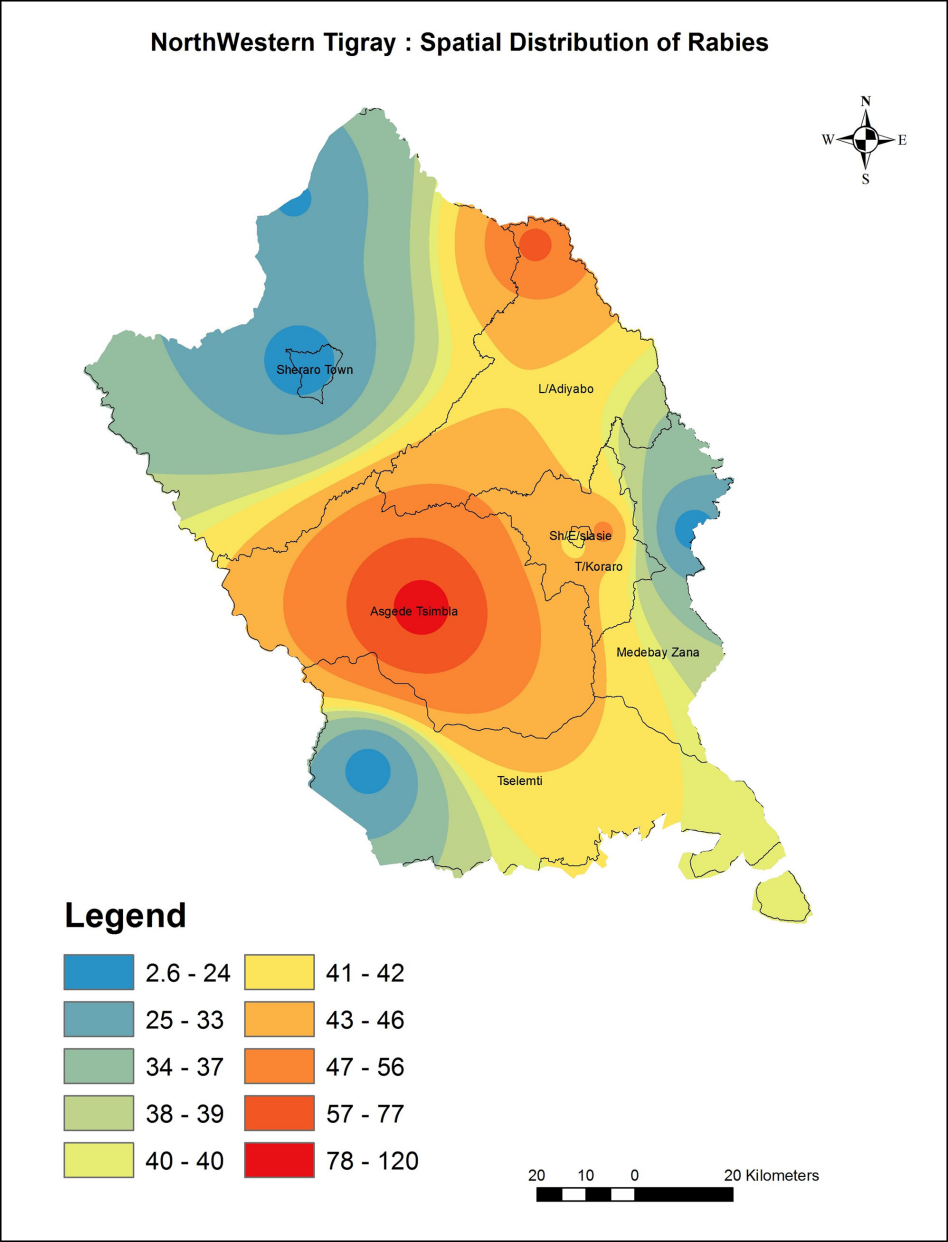
Spatial distribution of human rabies exposure.

**Figure 3:**
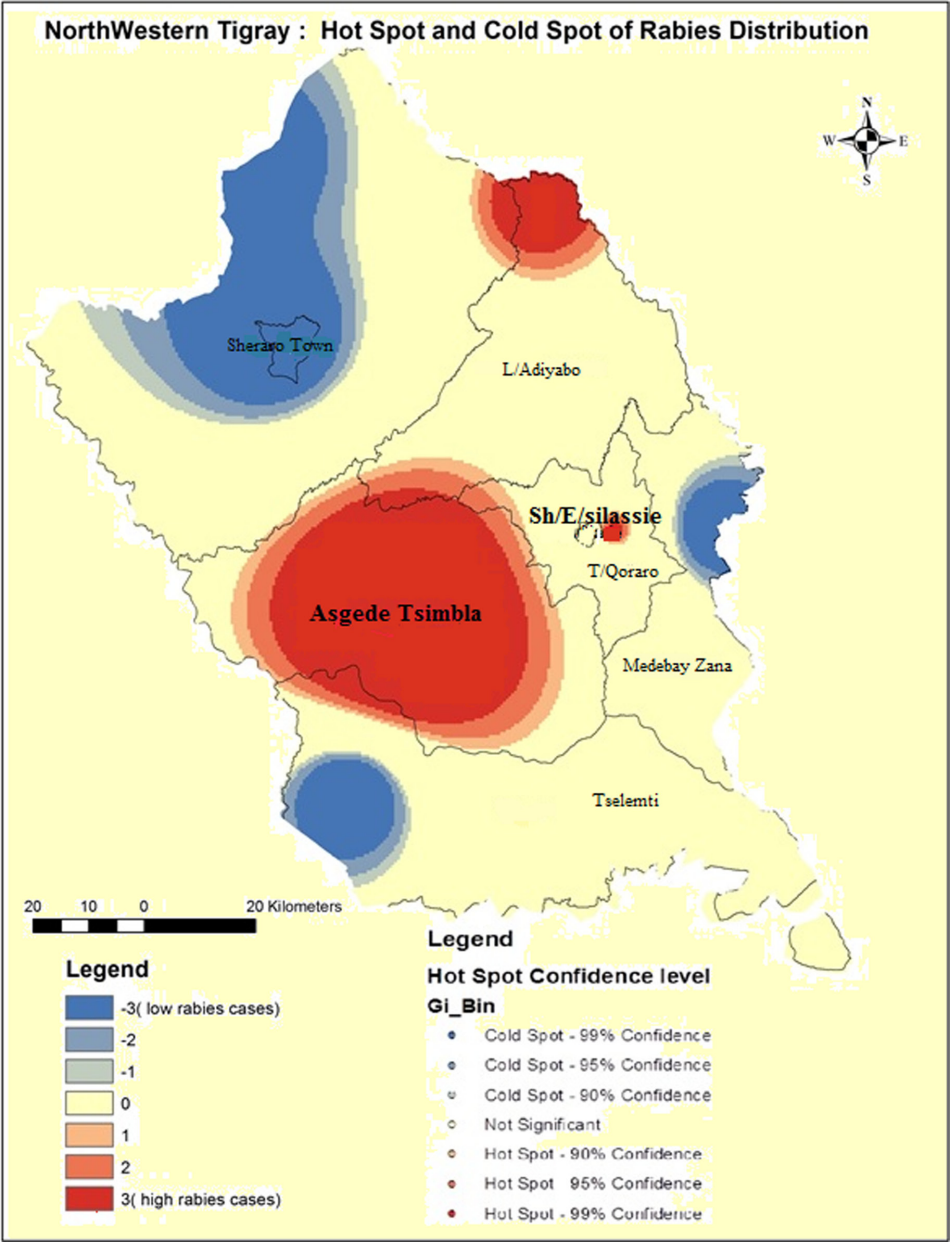
Hotspots and coldspots of human rabies exposure in Northwestern Tigray.

Our results showed that rabies exposure was distributed throughout in all districts of Northwestern Tigray. However, the spatial interpolation results indicated that the highest cluster of human rabies exposure were found in central part of Asgede Tsimbla, Shire Endaselassie and north eastern part of Laelay Adiabo which is indicated with a value ranging from 78–120 exposures. The lowest human rabies exposures were indicated in Sheraro, south west of Tselemti and Eastern part of Medebay Zana with a value ranging from 2–24 exposures (Figure 2). Similarly, results of hotspot analysis (Getis-Ord Gi statistic) of eight districts revealed that central parts of Asgede Tsimbla, Shire Endaselassie and north eastern part of Laelay Adiabo were the hot spots while Sheraro, south west of Tselemti and Eastern part of Medebay Zana districts were identified as cold spots of human rabies exposure during the study year. The red colored areas indicate statistically significant hotspots while steel blue areas represent significant cold spot areas (Figure 3).

**Figure 4:**
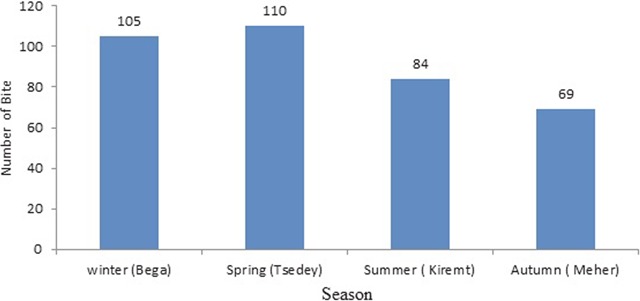
Human rabies exposure distribution by season in Northwestern Tigray, 2016.

**Figure 5:**
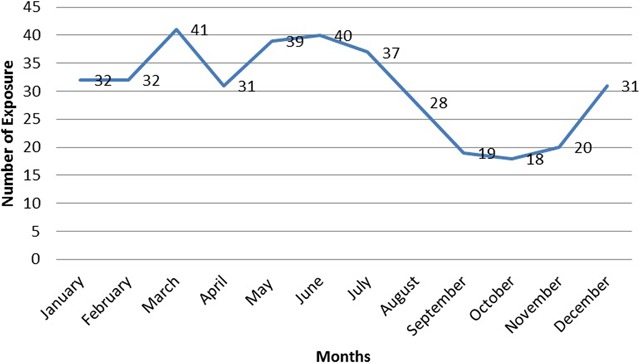
Month wise trend analysis of human rabies exposure distribution in Northwestern Tigray, 2016.

Season wise, the highest human rabies exposures were reported in Spring, in Amharic called *Tsedey* (April to June) followed by Winter in Amharic called *Bega* (January to March) while the lowest distribution of human rabies exposure was recorded in Autumn in Amharic called *Meher* (October to December) (Figure 4). The line graph (Figure 5) indicates the human rabies exposure distribution across months during the study year.

## Discussion

Dog mediated rabies is entirely preventable by vaccinating dogs and providing PEP to humans until dog rabies is eliminated. This necessitates conducting regular epidemiological surveillance programs to know the status of the disease in dogs and humans. Therefore, this research was conducted to investigate the epidemiological situation of human rabies in Northwestern Tigray so as to support local rabies intervention strategies in line with the Global Framework for the Elimination of Dog-Mediated Human Rabies, proposed by the World Health Organization (WHO), World Animal Health Organization (OIE), Food and Agriculture Organization of the United Nations (FAO) and Global Alliance for Rabies Control [14].

Our findings indicated that human rabies exposure during the study year was calculated to be 40 per 100,000 populations. This exposure was higher than previous studies reported from Ethiopia as well as other African and Asian countries [19,24,25,26,27,28]. The higher incidence in human rabies exposure may indicate that the existing control and prevention methods are not adequate and/or less functional. Other possible reasons which may contribute for the high incidence of rabies in the study area were discussed in our previous report [8]. Fahrion et al. have suggested that irresponsible dog ownership, excluding community engagement and failed public health and veterinary collaboration could make control of rabies unmanageable [10]. In contrast, the calculated incidence was lower than previously reported findings of four consecutive years (2012–2015) in the same study area [8]. This could be due to enhanced rabies intervention measures such as increasing mass dog vaccination campaigns than the previous years.

Results of this study revealed that individuals in the age range of 5–14 years were the most commonly exposed to rabies (43.2%; 95% CI, 38.2–48.3). Our finding was in line with previous studies which have been reported in Ethiopia as well as in Tanzania, Nigeria and Uganda, where children were at higher risk than adults [9,19,28,29,30,31,32]. On the contrary, findings from Azerbaijan indicated that adults were more commonly affected than children [33]. The explicitly shown difference in incidence among age groups may attribute to different reasons such as variations in study methods and socio-cultural differences among various communities. For instance, in some communities, adults may be at high risk of rabies due to the fact that they usually conduct their outdoor activities in distant places away from their home. On the other hand, children might be more commonly exposed to rabies in communities where their kids are not well attended. Besides, children have always a tendency to play with dogs and have low awareness as well as they frequently chase and/or throw stones, which can provoke dogs.

Gender wise, males (63%; 95% CI, 58.0–67.8) were at high risk than females (37%; 95% CI, 32.1–42.0), which was comparable with previous works [8,9,19,26,32,24,25]. This is probably due to the fact that most females are housewives, so they stay at home while males are engaged in outdoor activities. Moreover, it has been indicated that males had good knowledge on rabies (53.4%) and the tendency to prevent the disease than females (10.7%) [36].

In the present study, rural residents were more exposed to be bitten by rabid dogs than urban residents. This is also similar to other studies undertaken in different areas of Ethiopia as well as other Asian countries such as China, India and Iran [19,25,26,27,34,35]. As the population of unvaccinated dogs is higher in rural settings, unvaccinated dogs attack more rural people (82.9%) than urban people (3%). This could be due to low access of anti-rabies vaccines for dogs as well as poor awareness of rural residents not to vaccinate their dogs or poor flow information in the rural settings. Likewise, it has also been suggested that most farmers in the rural areas prefer to keep more than one dog to protect their animals from wild animal predators [8]. Jemberu et al. have suggested that the rise in dog population perhaps lead to higher infection rate [27]. Our results also showed that majority of the human rabies exposures (96.5%) were caused by unprovoked dogs, which had no history of vaccination (85.9%). In fact, lack of anti-rabies dog vaccine has been reported in Ethiopia [8,17,27]. Similarly, in Nigeria unvaccinated dogs were reported to cause 82.17% of the human bites [30].

During the study period, all human rabies exposures registered in the hospital were due to dog bites. Similar to earlier studies in Ethiopia and elsewhere, dog bite was the reason for most of human rabies exposures [9,17,19,26,27,29,37,38]. Two hundred ninety-six (80.4%) of the human rabies exposures were caused by stray dogs. This could be explained that stray dogs have a greater tendency of contracting the disease than owned and restricted ones. However, some reports in Nigeria and Iran have demonstrated that household dogs frequently bite and transmit rabies to humans [30,34,39]. The possible explanation for this might be household dogs were owned but unrestricted or free roaming, which can frequently contact with neighboring dogs. It was previously reported that owned dogs which wander freely were found to be more exposed to rabies than owned and controlled dogs [38].

Result of this study also demonstrated that higher dog bite injuries (98.1%) were reported on limbs. This was concordant with previous findings and it could be attributed to frequent use of legs and arms to defend from aggressive dogs [17,25,34,35,40]. In the present study, the proportion of type II exposure (56.8%) was higher than that of type III (43.2%). This was consistent with other reports from Iran that the majority of the dog bite injuries were small and superficial lesions [34,35].

The spatial interpolation results indicated that the highest cluster of human rabies exposures were found in central part of Asgede Tsimbla, Shire Endaselassie and north eastern part of Laelay Adiabo, which is indicated with a value ranging from 78–120 exposures. These were identified as hotspots for human rabies. While Sheraro, south west of Tselemti and Eastern part of Medebay Zana had 2–24 exposures per district and these were identified as coldspots (Figures 2 and 3). This refers that rabies exposures predominantly occur in these specific hotspot districts. This could be explained by the existence of high population of dogs in these districts or increased public awareness about the disease. Lee et al. have suggested that the geographic aggregation of rabies exposures could also attributed to better economic returns of the societies to receive PEP [41]. Other studies explain this could be due to low coverage of anti-rabies dog vaccination programmes (lower than 20%) [42,43]. However, data regarding the coverage of anti-rabies dog vaccination staus is scarce in the area. The existence of wildlife population in the hotspot areas could also be a contributing factor leading to spillover infection in pet animals (particularly dogs) and inflicting subsequent bites in humans [3,41,44,45]. However, the role of wildlife in rabies transmission cycle in the study area is not yet explored. This necessitates implementing an in-depth investigation to explore the specific socio-economic factors and dog population census as well as the possible role of wildlife in the transmission cycle of rabies in these areas.

Season wise, the highest human rabies exposure exposures were reported in Spring, followed by Winter while the lowest distribution of human rabies was recorded to occur in Autumn (Figure 4). Strong peaks of human rabies exposure per month during the study year were recorded between March and July (Figure 5). These months (particulaly winter and spring seasons) are usually characterized by hot and dry weather conditions. Our finding was concordant with reports from other Asian countries: Lao People’s Democratic Republic, Veitnam and Buhtan that high incidence of rabies occurr during the dry seasons [41,45,46]. This could be associated with seasonal dog-breeding cycles. This biological phenomena of dogs could increase the prevalence of rabies due to increased contacts because of fights/bites among them leading to increased transmission of the virus [47,48]. Seaonality of rabies has also been widely described in other countries: Veitnam, Peru, the United States, Chile, Bolivia and Buhtan [41,45,47,49,50,51,52].

The study had its own limitations. The research was a health facility based study. Data of human rabies exposures were collected from Suhul general hospital that originated from eight districts (refer the descreption of the study area) and all the residents of the adminstrative zone were considered as population at risk. However, we are unable to find detailed population census categorized based on age, sex and residence. Without this categorization we could not procceed to further analyses such as multiple logistic regression. Moreover, the study was solely based on human PEP data and the laboratory confirmation of brain samples of suspected dogs was not practiced in the hospital due to a lack of rabies diagnostic facilities.

## Conclusion

The present study provided basic epidemiological information on the potential risk factors associated with human rabies exposure. Moreover, our findings provided basis for understanding the spatial and seasonal patterns of human rabies in Northwestern Tigray districts for the first time. As recommendation, an in-depth investigation should be undertaken to explore the cultural and socio-economic factors associated with increased risk of human rabies as well as the possible role of wildlife in the transmission cycle of rabies in these areas.
